# Supplementary material for: Pore evolution mechanisms during directed energy deposition additive manufacturing
Source: Nat Commun. 2024 Feb 24;15:1715. doi: 10.1038/s41467-024-45913-9 (PMC10894260; doi:10.1038/s41467-024-45913-9)
Supplement: Supplementary file 1 — Supplementary Information [file 41467_2024_45913_MOESM1_ESM.docx]

# Supplementary Information

**Pore evolution mechanisms during directed energy deposition additive manufacturing**

Kai Zhang^1,2,*^, Yunhui Chen^1,2,3,4^, Sebastian Marussi^1,2^, Xianqiang Fan^1,2^, Maureen Fitzpatrick^1,3^, Shishira Bhagavath^1,2^, Marta Majkut^3^, Bratislav Lukic^3^, Kudakwashe Jakata^3,5^, Alexander Rack^3^, Martyn A. Jones^6^, Junji Shinjo^7^, Chinnapat Panwisawas^8^, Chu Lun Alex Leung^1,2^, Peter D. Lee^1,2, *^

^1^ Mechanical Engineering, University College London, London, WC1E 7JE, UK

^2^ Research Complex at Harwell, Harwell Campus, Didcot, OX11 0FA, UK

^3^ ESRF- The European Synchrotron, Grenoble, 38000, France

^4^ School of Engineering, RMIT University, Melbourne, VIC 3000, Australia

^5^ Diamond Light Source, Harwell Campus, Oxfordshire, OX11 0DE, UK

^6^ Rolls-Royce plc, PO Box 31, Derby, DE24 8BJ, UK

^7^ Next Generation Tatara Co-Creation Centre, Shimane University, Matsue, 690-8504, Japan

^8^ School of Engineering and Materials Science, Queen Mary University of London, London, E1 4NS, UK

* Corresponding authors: [kai-zhang@ucl.ac.uk](mailto:kai-zhang@ucl.ac.uk) ; [peter.lee@ucl.ac.uk](mailto:peter.lee@ucl.ac.uk)

**This file includes:**

- Supplementary Method 1
- Supplementary Figures 1-15
- Supplementary Discussions 1-2
- Supplementary Tables 1-3
- Supplementary References

**Other Supplementary Files for this manuscript include:**

- Supplementary Movies 1-9
- Description of Additional Supplementary Files

## Supplementary Method 1

**Multiphysics modelling during direct energy deposition.**

As shown in **Methods**, the fluid flow equations of mass, momentum and temperature are solved in fully coupled fashion ^1,2^, which are

(mass) $\frac{\partial\rho}{\partial t}+(\boldsymbol{u}\cdot\nabla)\rho=-\rho\nabla\cdot\boldsymbol{u}$ (1)

(momentum) $\frac{\partial\boldsymbol{u}}{\partial t}+(\boldsymbol{u}\cdot\nabla)\boldsymbol{u}=-\frac{\nabla p}{\rho}+\boldsymbol{Q}_{\boldsymbol{u}}+\boldsymbol{g}+\boldsymbol{F}_{\boldsymbol{u},surf}$ (2)

(temperature) $\frac{\partial T}{\partial t}+(\boldsymbol{u}\cdot\nabla)T=-\frac{p\nabla\cdot\boldsymbol{u}}{\rho c_{p}}+Q_{T}$ (3)

where *ρ* is the density, **u** is the velocity, *T* is the temperature, *p* is the pressure and *c_p_* is the constant-pressure heat capacity. **Q_u_** represents the Newtonian viscous force and Darcy’s force in the mushy zone, given by

$Q_{u,i}=\frac{1}{\rho}\frac{\partial\tau_{ij}}{\partial x_{j}}-\frac{\nu}{K}\left( \frac{(1-f_{L})^{2}}{{f_{L}}^{3}} \right)u_{i},\quad\tau_{ij}=\mu\left( \frac{\partial u_{i}}{\partial x_{j}}+\frac{\partial u_{j}}{\partial x_{i}} \right)-\frac{2}{3}\mu\nabla\cdot\boldsymbol{u}\delta_{ij}$ (4)

where *K* is the permeability coefficient related to grain scale, *f_L_* is the liquid fraction determined by the temperature, *ν* is the kinematic viscosity and *μ* is the dynamic viscosity. **g** is the gravitational acceleration. **F_u_**_,_*_surf_* represents the interfacial surface tension force and the Marangoni effect, formulated as

$\boldsymbol{F}_{s}=\sigma\kappa\delta\boldsymbol{n}+\left( \nabla\sigma-\left( \nabla\sigma\cdot\boldsymbol{n} \right)\boldsymbol{n} \right)\delta$ (5)

where *σ* is the temperature-dependent surface tension coefficient, *κ* is the local surface curvature and **n** is the unit vector normal to the surface. *δ* is non-zero only on the surface. *Q_T_* represents the heat transport, including heat conduction by Fourier’s law, enthalpy transport by mass diffusion, viscous work, latent heat for phase change and radiation, given as

$Q_{T}=\frac{1}{\rho c_{p}}\left[ \nabla\cdot\left( \lambda\nabla T \right)+\frac{\partial\tau_{ij}u_{i}}{\partial x_{j}}-\frac{D\rho\Delta h}{Dt} \right]+\varepsilon\sigma_{SB}(T^{4}-{T_{0}}^{4})\left| \nabla\varphi\right|$ (6)

where Δ*h* is the latent heat. Radiation is included on the heated metal surface identified by the gradient of the interface-identifying colour function $\left| \nabla\varphi\right|$, with *σ_SB_* the Stefan-Boltzmann constant and *ε* the emissivity. The laser power is given to the melt pool surface by the ray tracing method and the absorbed heat is given on the surface as a source term. The physical properties such as viscosity and thermal conductivity are derived as in the reference ^2^.

The surface capturing is done by the Coupled Level-Set/Volume-Of-Fluid (CLSVOF) method to assure shape accuracy and volume conservation. The level-set function (signed distance function from the surface) is governed by

$\frac{\partial F}{\partial t}+(\boldsymbol{u}\cdot\nabla)F=\left| \nabla F \right|\frac{dN}{dt}$ (7)

where ${dN}/{dt}$ is the surface growth speed due to metal deposition and is estimated from the powder feeding rate. By converting *F* into $\varphi$, where $\varphi$ ($0\leq\varphi\leq1$) is a Heaviside function of *F*, the density at the melt pool surface, for example, is given by $\rho=\left( 1-\varphi\right)\rho_{G}+\varphi\rho_{L}$. *ρ_L_* and *ρ_G_* are the liquid and gas density, respectively.

For bubbles, two tracking methods are used, Eulerian interface tracking and Lagrangian point particle tracking. The appropriate regime of bubble modelling can be clarified by the particle Reynolds number, the particle Weber number and the Stokes time scale. The Reynolds number is given by

$Re=\frac{\rho UD}{\mu}$ (8)

where *ρ* is the liquid density, *D* is the bubble diameter and *μ* is the liquid viscosity. *U* is the slip velocity magnitude between the bubble and the surrounding liquid flow. The Weber number is given by

$We=\frac{\rho U^{2}D}{\sigma}$ (9)

where *σ* is the surface tension coefficient. The Stokes time scale is given by

$\tau=\frac{\rho D^{2}}{18\mu}$ (10)

which gives the relaxation time scale for the bubble to follow the outer liquid flow (namely, the slip velocity becomes negligibly small). The point particle assumption is justified when the flow around the bubble is very slow and symmetric (*Re*<1, for example) and the Stokes drag law can be used (*τ* is smaller than the flow time scale) and the bubble shape is spherical due to sufficiently stronger surface tension than the inertial force (*We*<0.1, for example).

Taking the lower limits of Reynolds number of 1.0, the Weber number of 0.1 and the Stokes time scale of 0.1 ms (from the experimental observations), the regime map becomes as shown in Supplementary Fig. 15. The region below each line represents the point particle justification for each parameter and the bottom-left region (green region) satisfies all the conditions. The small bubbles tracked in the experiment, which are mostly pre-existing bubbles from the feedstock, lie in this regime of the map and the point particle assumption is justified for these bubbles in the simulation (the method is described in the next paragraph). However, for the coalesced large bubbles, the slip flow velocity is ~0.3-0.4 m s^-1^ (max) and they are out of the point particle regime. In this case, the numerical modelling needs to resolve the shape of a finite-volume bubble, and Eulerian interface tracking by Eq. (7) is used.

For the small bubble tracking cases in Fig. 8b and 8d-e, the bubbles are treated as Lagrangian point particles, whilst in other cases, the bubble surface shape is captured by the level-set method. The Lagrangian equations of motion, temperature and mass change are

$\frac{d\boldsymbol{x}_{p}}{dt}=\boldsymbol{u}_{p}$, (11)

$m_{p}\frac{d\boldsymbol{u}_{p}}{dt}={m_{p}f_{1}}/\tau\cdot(\boldsymbol{u}_{f}-\boldsymbol{u}_{p})$, (12)

$m_{p}c\frac{dT_{p}}{dt}={(m_{p}Nu}/{3\tau_{p}Pr})\cdot c_{p,f}f_{2}(T_{f}-T_{p})+\dot{m}_{p}L$ (13)

$\frac{dm_{p}}{dt}=-({m_{p}}/\tau)\cdot({Sh}/{3Sc})\cdot\ln(1+B_{M})$ (14)

The subscript *p* denotes particles (bubbles) and *f* represents the flow around the particles. **x**_p_ is the particle position, **u**_p_ is the particle velocity and *m_p_* is the particle mass. $f_{1}={C_{D}Re}/{24}$ is the Stokes drag for low velocity and the particle Reynolds number is given by Eq. (8) and the Stokes relaxation time scale is defined by Eq. (10).

In the powder temperature equation, *c* is the heat capacity, *Nu* and *Pr* are the Nusselt and Prandtl numbers, respectively, and *Nu* is determined by the Ranz-Marshall correlation. $f_{2}=\beta/{(\exp\beta-1)}$ with $\beta=-{1.5Pr\dot{m}_{p}\tau}/{m_{p}}$ and *L* is the latent heat of the material. In the mass transfer equation due to evaporation, *Sh* is the Sherwood number determined by the Ranz-Marshall correlation. The Spalding transfer number by mass fraction *Y* is $B_{M}={(Y_{V,s}-Y_{V,f})}/{(1-Y_{V,s})}$, where *s* denotes the surface. The surface vapour mass fraction is modelled by the Clapeyron-Clausius relation.

The numerical scheme is based on CIP (Cubic Interpolated Pseudo-particle or Constrained Interpolation Profile) method, in which a third-order polynomial fitting is used for flow variables and their derivatives to assure the accuracy. Further details of the numerical method can be found in ^1,2^. Here, two grid resolutions are used. For the basic flow and bubble coalescence cases (Fig. 6, 7a, Supplementary Fig. 8, 9, 10, 11) the resolution is 16 μm, and for the bubble trajectory tracking cases (Fig. 7b, c, 8b, d, e, Supplementary Fig. 12 and 13) it is 32 μm. The latter resolution is used to simulate the longer time behaviour and it is confirmed that this resolution can also reproduce the same flow structures in the melt pool.

## Supplementary Figures


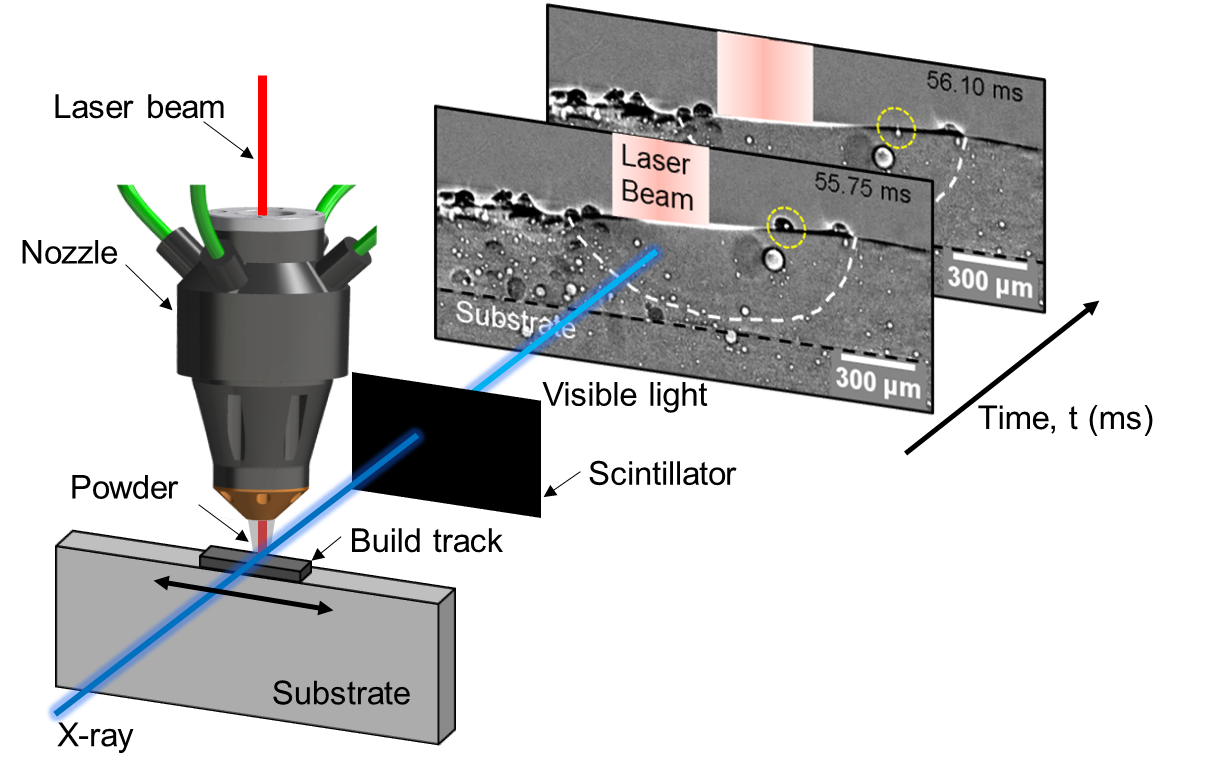


Supplementary Fig. 1. Schematic of the *in situ* synchrotron X-ray imaging experiment of directed energy deposition additive manufacturing (DED AM) with time-series radiographs acquisition. A bubble formed from the powder marked with a yellow circle. All white scale bars correspond to 300 µm.


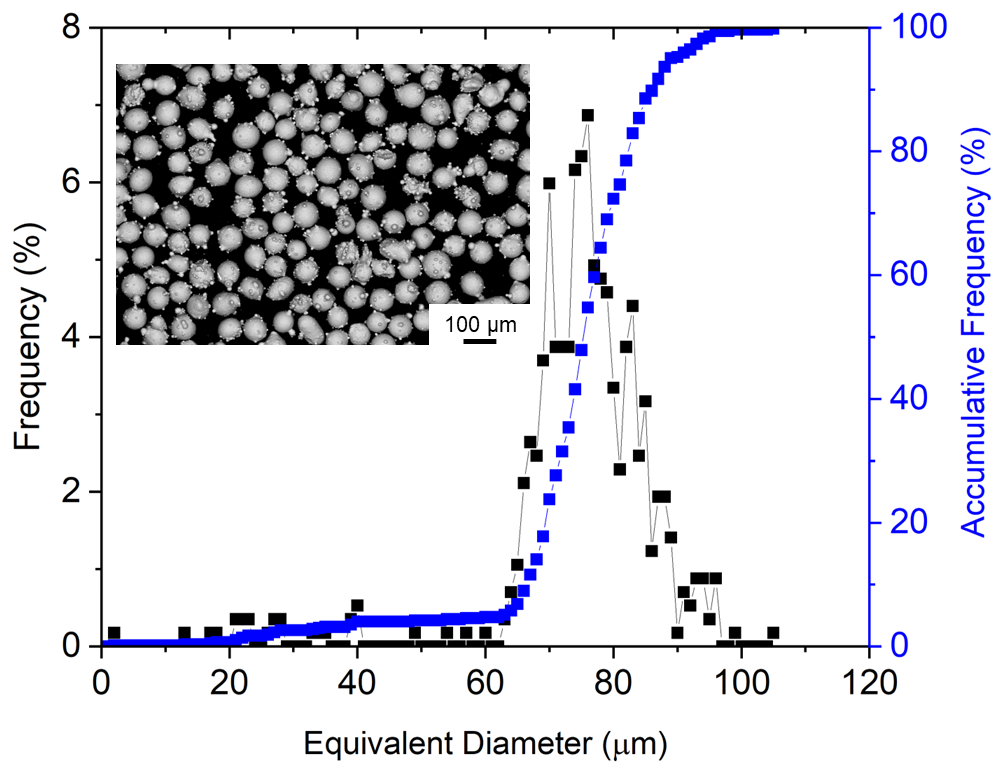


Supplementary Fig. 2. Size distribution of RR1000 nickel based superalloy powder. The inset shows the backscatter scanning electron image of RR1000 powder. The powder particles were segmented using Otsu’s method and separated by the watershed algorithm in MATLAB (MathWorks, USA).


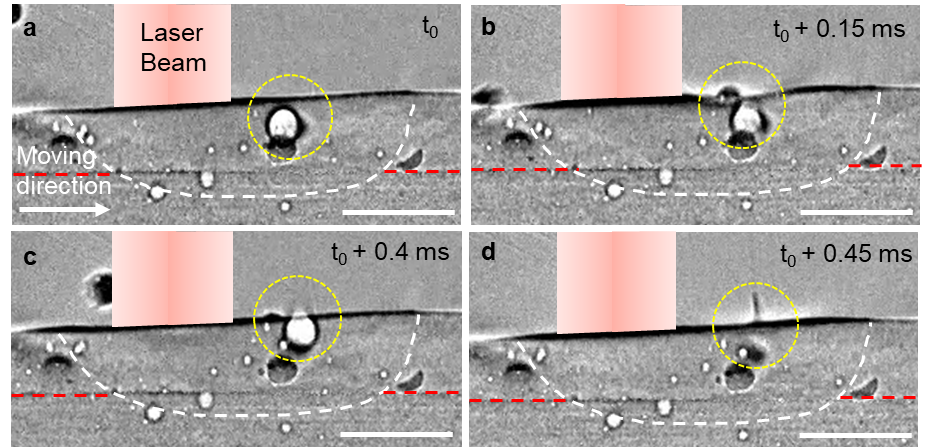


Supplementary Fig. 3. **a-d** A large bubble escape from the melt pool with a high-speed synchrotron X-ray imaging. The laser power is 160 W, the traverse speed is 2 mm s^-1^ and layer 3. (Scale bars are 300 μm).


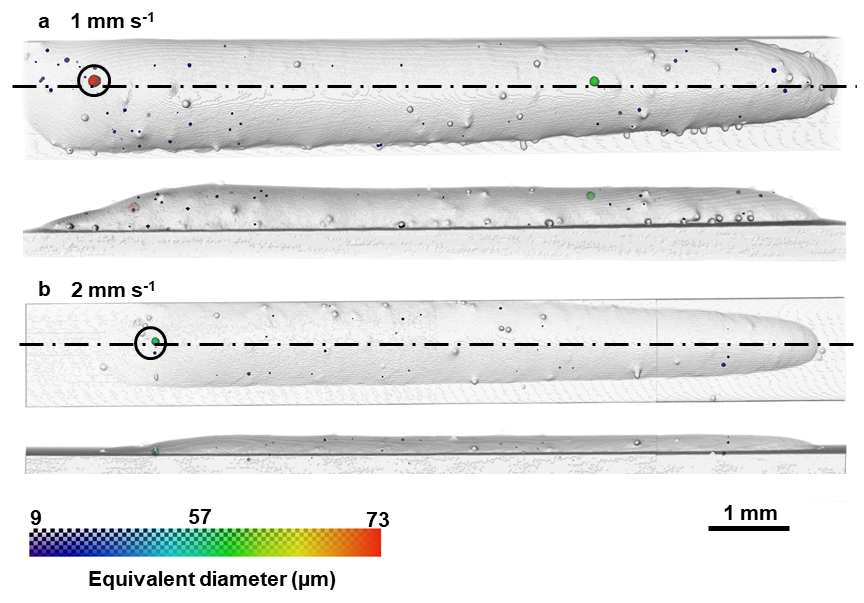


Supplementary Fig. 4. Tomography of DED tracks at **a** 1 mm s^-1^ and **b** 2 mm s^-1^. The laser power is 160 W, layer 3. The large pore at the end of the track was marked with a black circle. The scale bar is 1 mm.


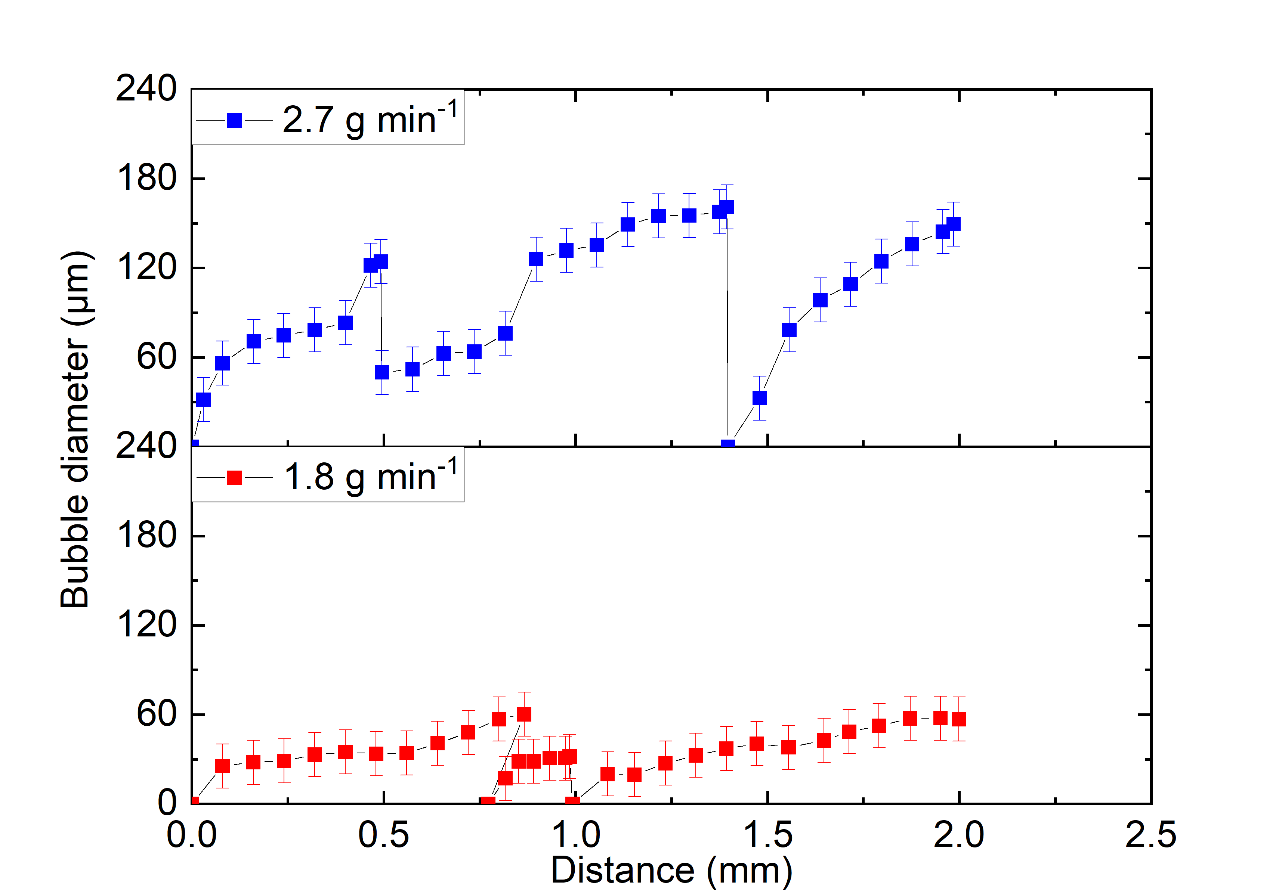


Supplementary Fig. 5. Bubble diameter with increasing distance in different powder flow rates (1.8 and 2.7 g min^-1^). The laser power is 150 W and the traverse speed is 1 mm s^-1^, layer 1. The bubble diameter error bars are calculated as ±2 pixels, equivalent to the segmentation uncertainty.


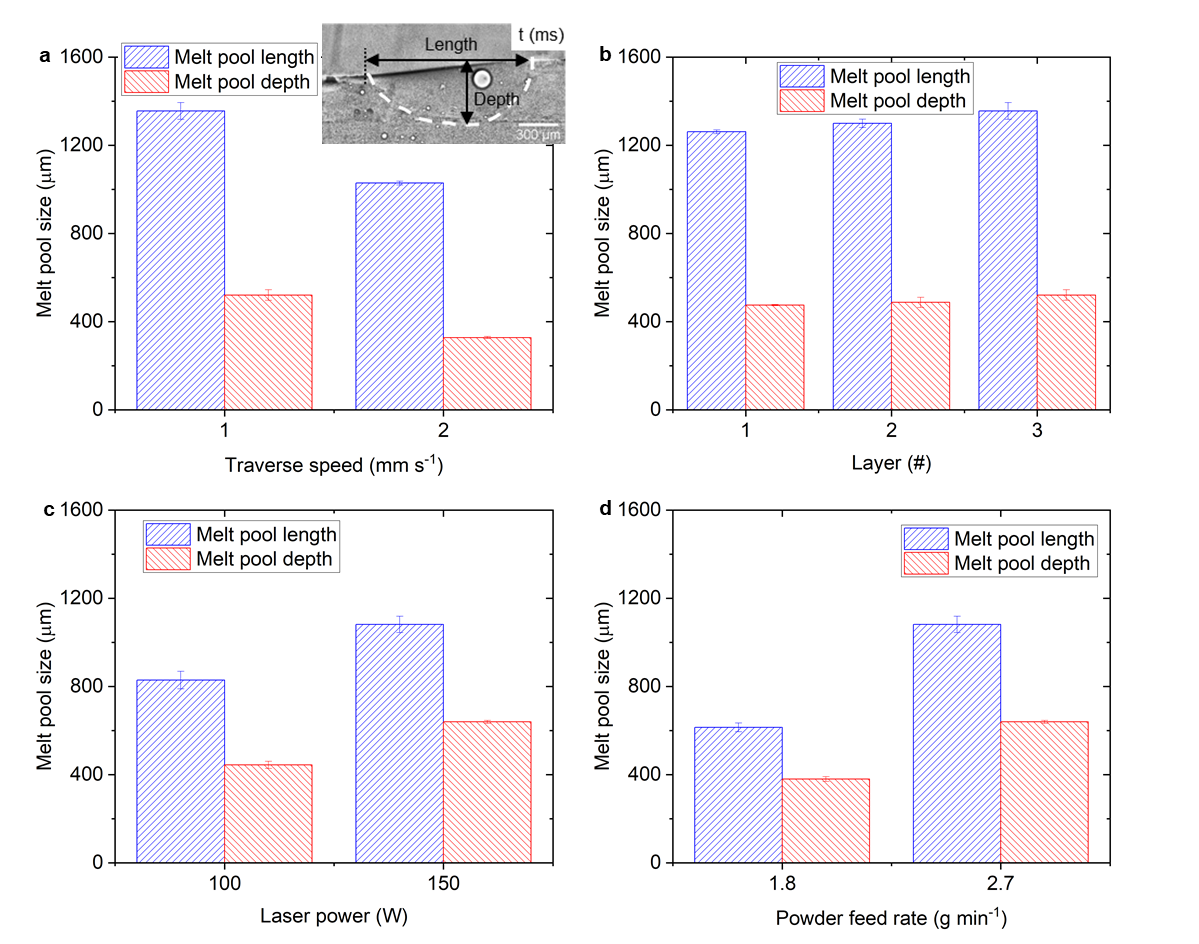


Supplementary Fig. 6. Melt pool length and depth as a function of **a** traverse speed, **b** layer, **c** laser power and **d** powder feed rate. The error bars represent standard deviation. The inset in **a** shows the radiograph indicating the melt pool length and depth.


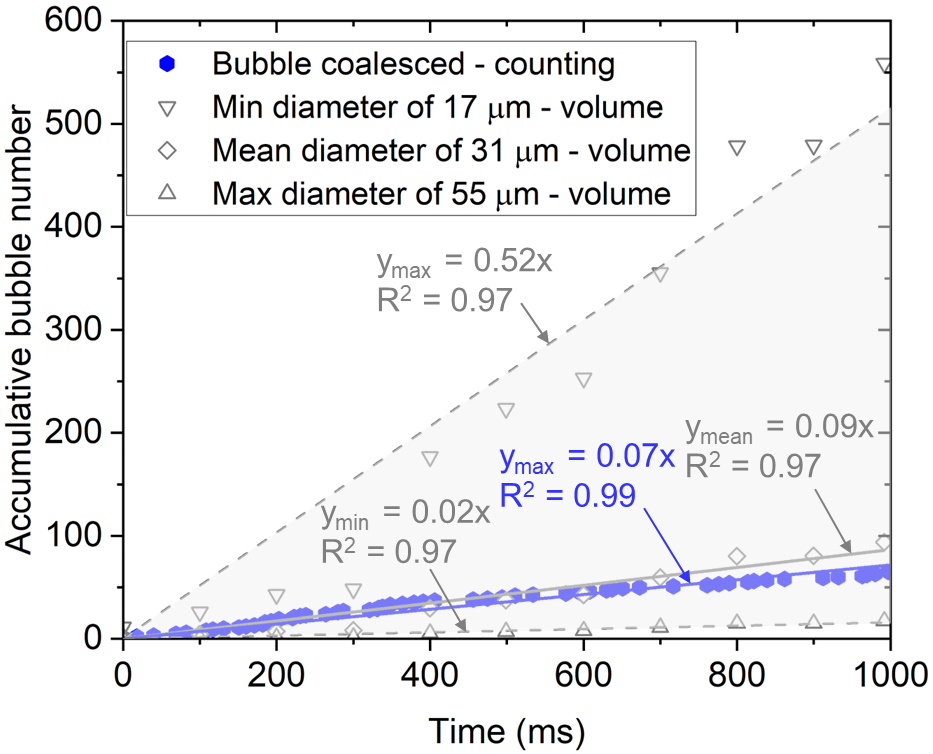


Supplementary Fig. 7. Accumulative coalesced bubble number with increasing time. The blue solid symbol represents the bubble number for coalescence by counting. The maximum, mean and minimum bubble number for coalescence is calculated using the volume of the large coalesced bubble divided by the volume of the initial bubble with the minimum, mean and maximum diameters of 17, 31 and 55 µm, respectively. The laser power is 160 W, and the traverse speed is 1 mm s^-1^, layer 3.


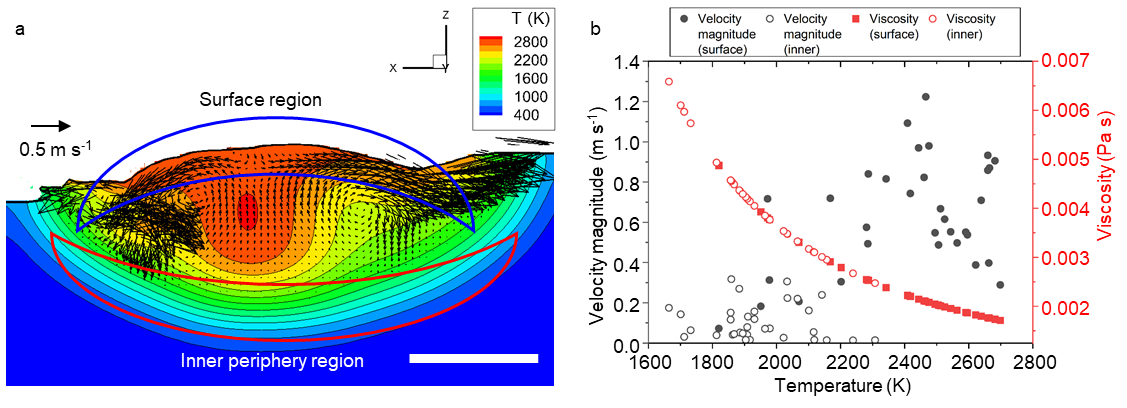


Supplementary Fig. 8. Modelling results showing velocity magnitude and viscosity in the melt pool. **a** regions of measurement. T in colour bar represents temperature in K. The scale bar is 300 μm. **b** velocity magnitude (m s^-1^) and viscosity (Pa s). The open symbols denote the deep inner region of the melt pool, and the closed symbols represent the surface region.


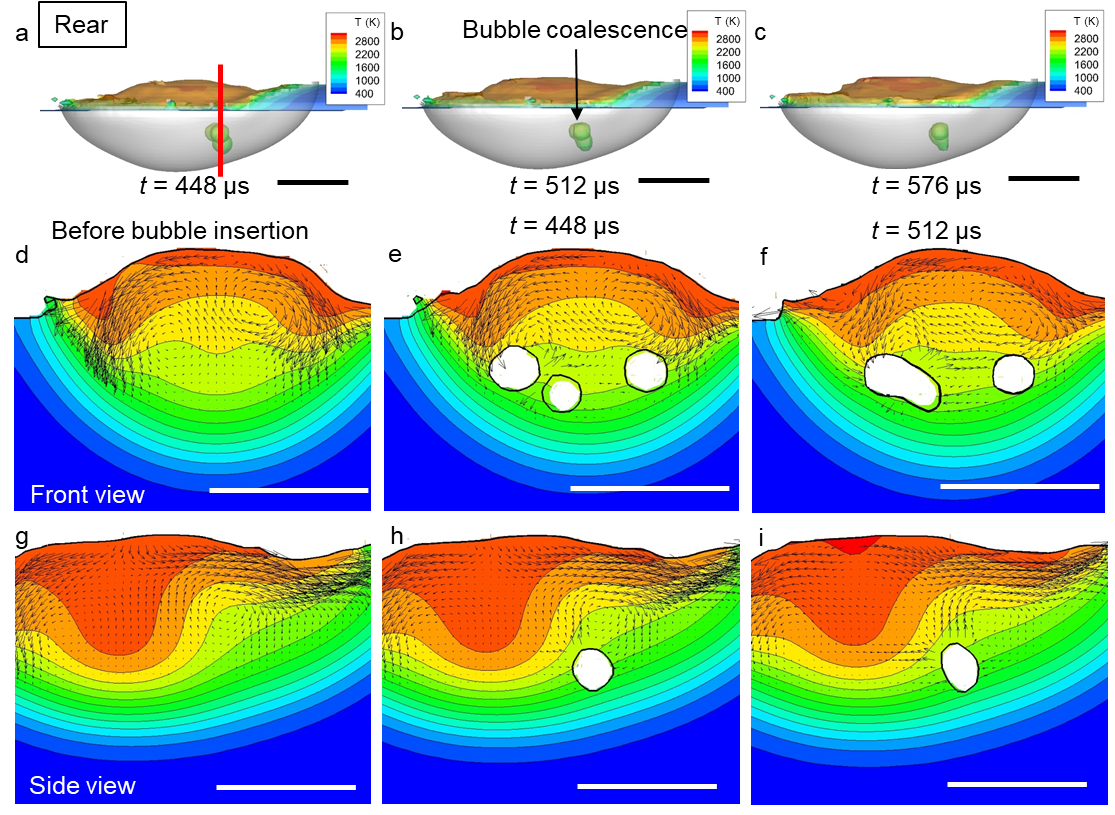


Supplementary Fig. 9. Modelling results showing bubble coalescence in the rear/back of the melt pool. 3D views of bubbles in rear location **a** at *t* = 448 µs, **b** bubble coalescence at *t* = 512 µs, and **c** *t* = 576 µs. Front views of **d** before bubble insertion, **e** at *t* = 448 µs, **f** bubble coalescence at *t* = 512 µs. Side views of **g** before bubble insertion, **h** at *t* = 448 µs, **i** bubble coalescence at *t* = 512 µs. T in the colour bar represents temperature in K. (Scale bars are 300 μm).


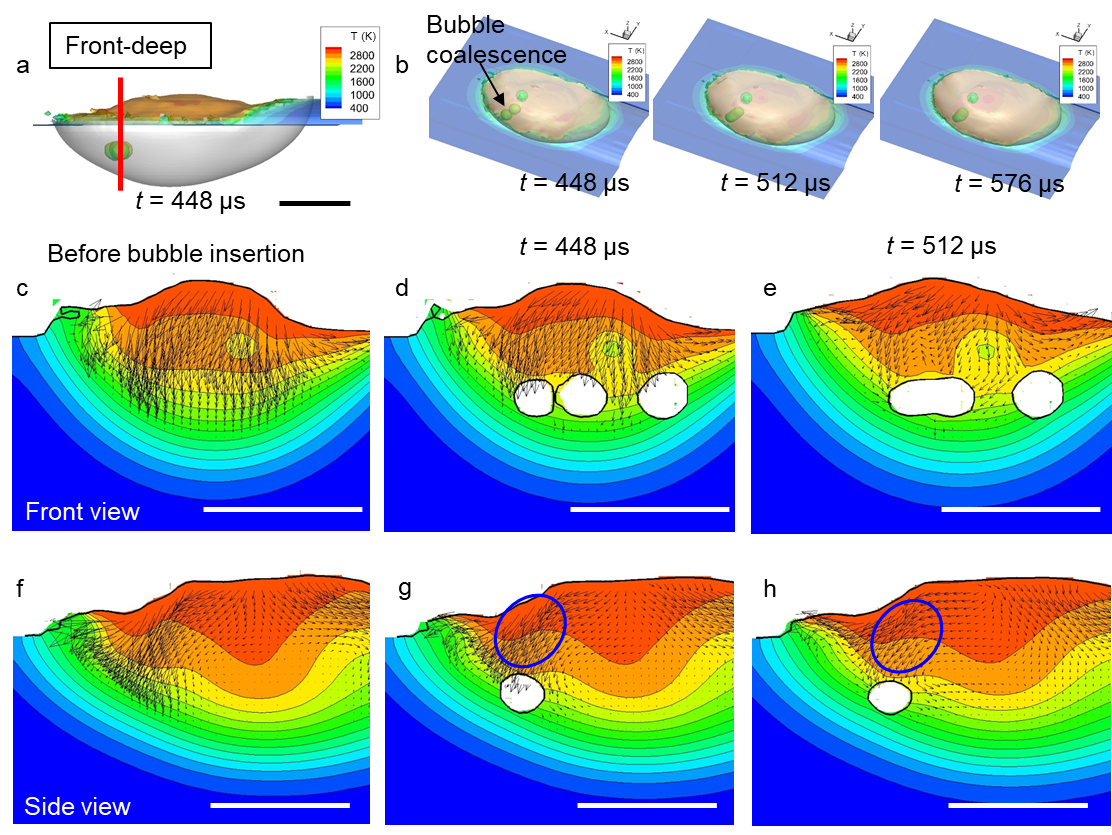


Supplementary Fig. 10. Modelling results showing bubble coalescence in the front-deep of the melt pool. **a** 3D views of bubbles in front-deep location at *t* = 448 µs, **b** 3D views of bubble coalescence at *t* = 448 µs, 512 µs and 576 µs. Front views of **c** before bubble insertion, **d** at *t* = 448 µs, **e** bubble coalescence at *t* = 512 µs. Side views of **f** before bubble insertion, **g** at *t* = 448 µs, **h** bubble coalescence at *t* = 512 µs. T in the colour bar represents temperature in K. (Scale bars are 300 μm).


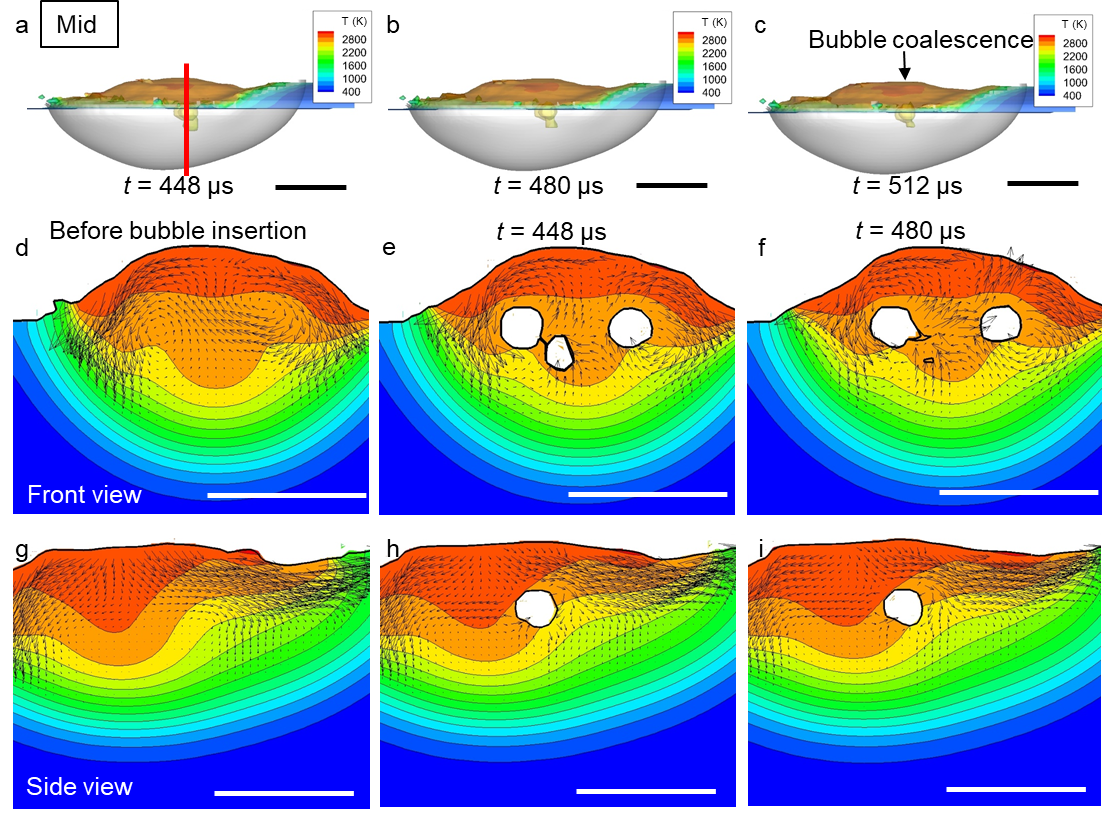


Supplementary Fig. 11. Modelling results showing bubble coalescence in the middle of the melt pool. 3D views of bubbles in middle location **a** at *t* = 448 µs, **b** bubble coalescence *t* = 480 µs, and **c** *t* = 512 µs. Front views of **d** before bubble insertion, **e** *t* = 448 µs, **f** bubble coalescence *t* = 480 µs. Side views of **g** before bubble insertion, **h** *t* = 448 µs, **i** bubble coalescence *t* = 480 µs. T in the colour bar represents temperature in K. (Scale bars are 300 μm).


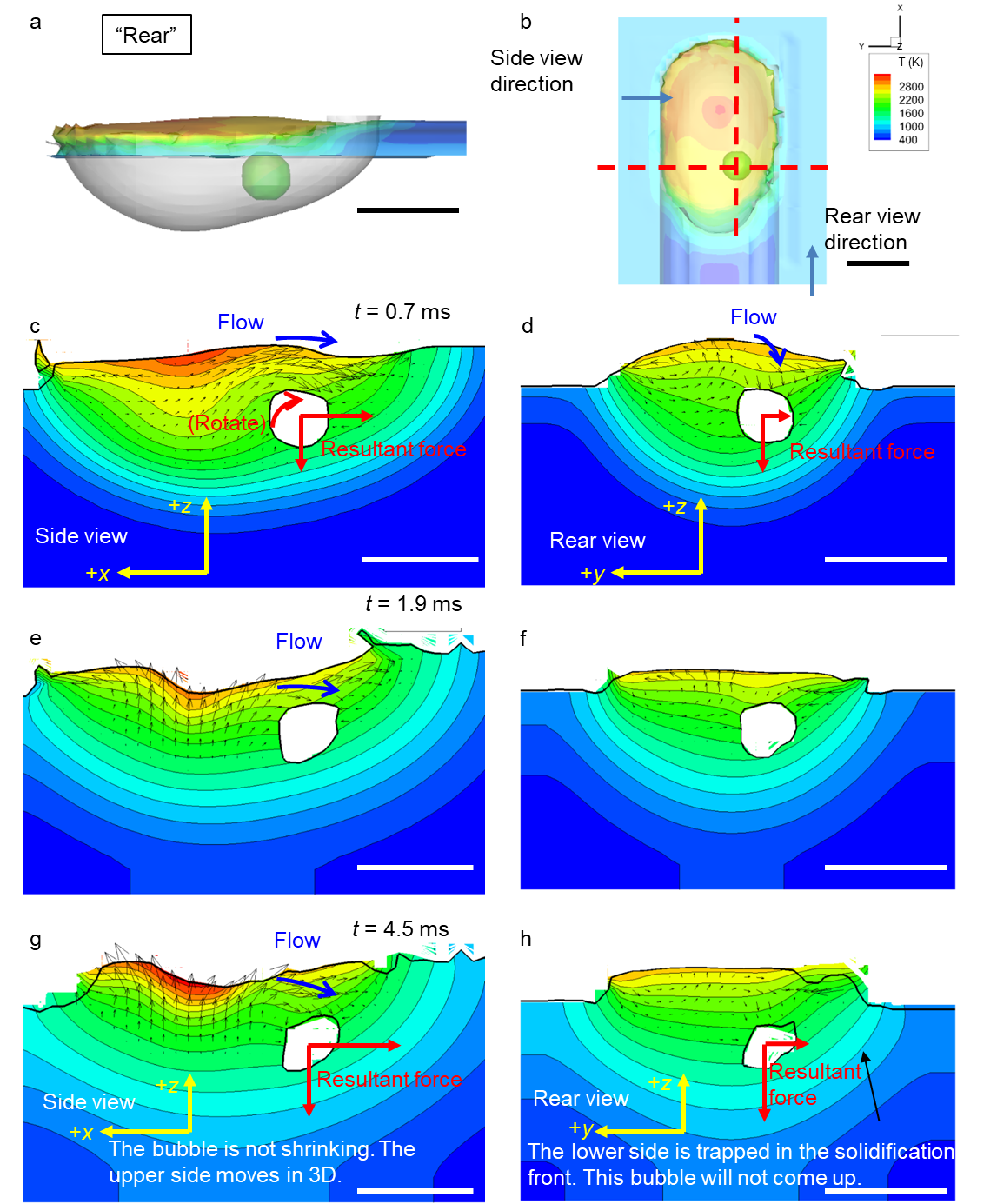


Supplementary Fig. 12. Modelling results showing bubble pushing. **a** 3D view and **b** top view of a large bubble in a rear/back location. **c** side view and **d** rear view of a large bubble pushed in melt pool at *t* = 0.7 ms from bubble insertion *t* = 0 ms. **e** side view and **f** rear view of bubble pushed in melt pool at *t* = 1.9 ms. **g** side view and **h** rear view of bubble trapped in solidification front at *t* = 4.5 ms. T in the colour bar represents temperature in K. (Scale bars are 300 μm). Force direction and magnitude are indicated with red arrows in **c**, **d, g** and **h**.


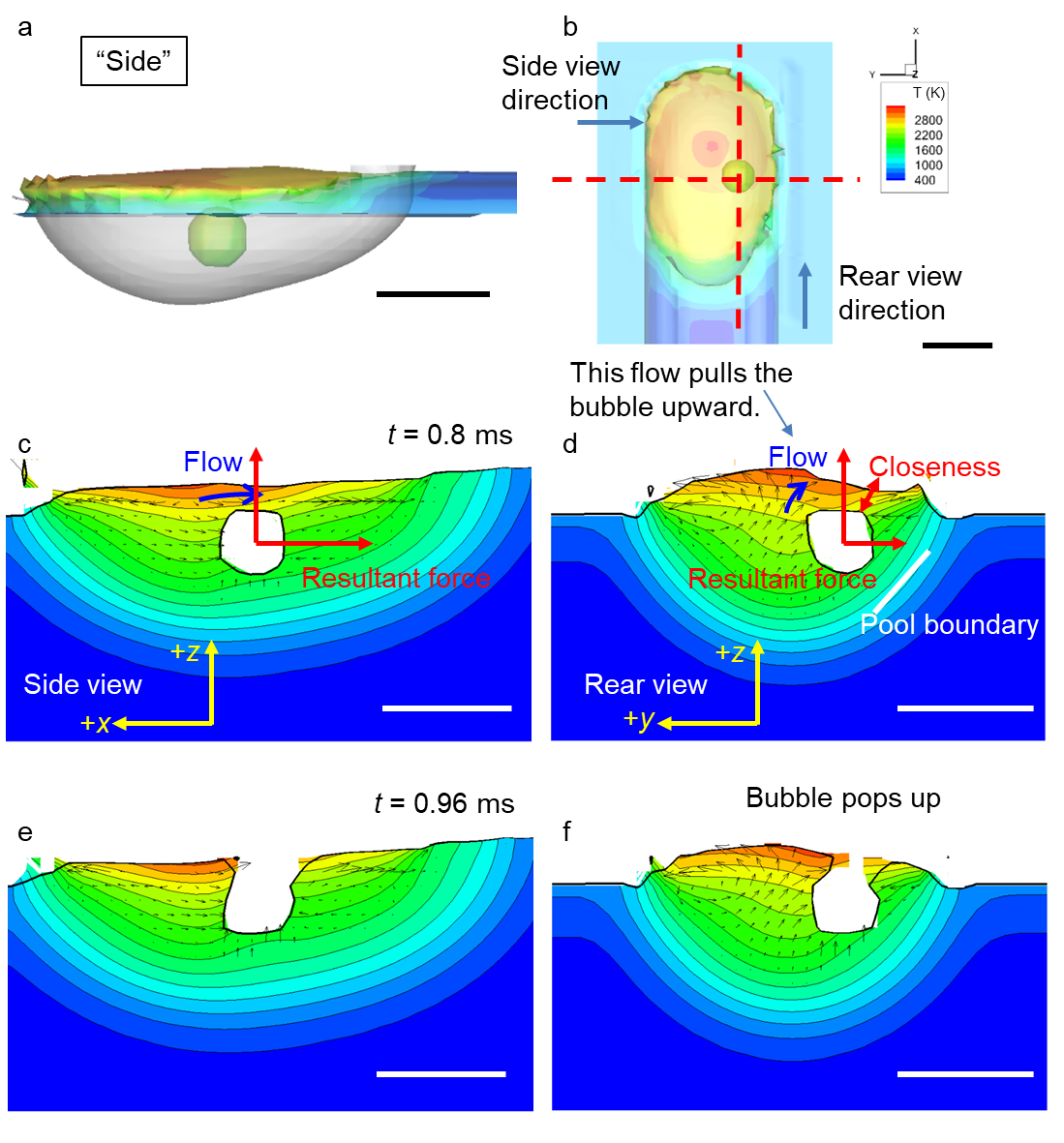


Supplementary Fig. 13. Modelling results showing bubble pop up. **a** 3D view and **b** top view of a large bubble in a side location. **c** side view and **d** rear view of a large bubble pushed in melt pool at *t* = 0.8 ms from bubble insertion at *t* = 0 ms. The corresponding schematic of force directions in the bubble pop up case. Force direction and magnitude are indicated by the red arrows in **c** and **d**. **e** side view and **f** rear view of bubble pop up at *t* = 0.96 ms. T in the colour bar represents temperature in K. (Scale bars are 300 μm).


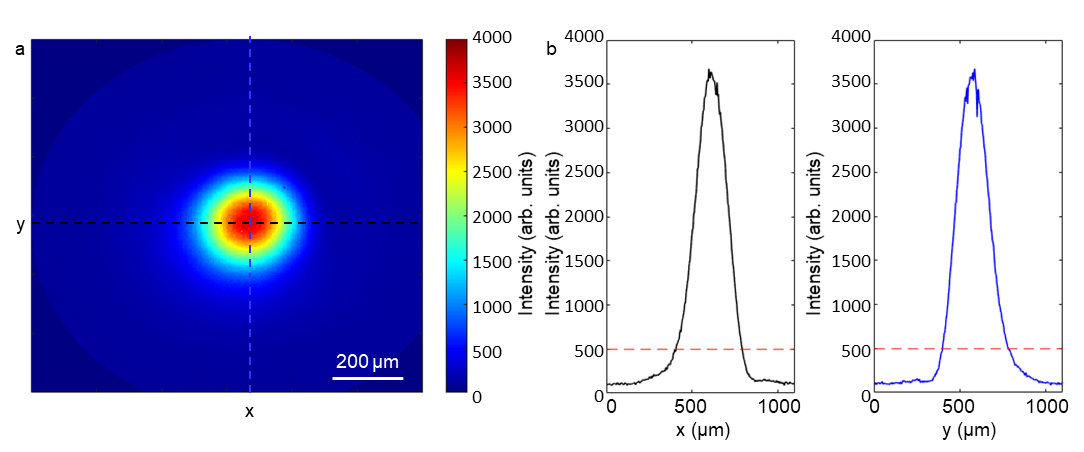


Supplementary Fig. 14. **a** The intensity colourmap of laser beam profiling. **b** the laser beam profile fitted by a Gaussian fitting. The white scale bar corresponds to 200 µm.


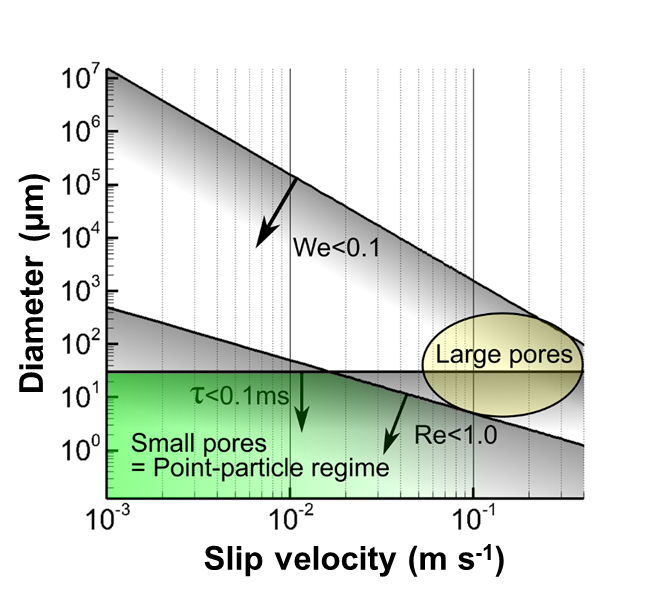


Supplementary Fig. 15. Bubble (pore) modelling regime. The green region in the bottom-left is the Lagrangian point particle regime. The experimentally observed large (coalesced) bubbles are in the yellow region where Eulerian interface tracking is needed.

## Supplementary Discussion 1

As shown in Supplementary Table 2, the melt pool length (500 - 2000 µm) and depth (300 - 1200 µm) cover the most range of the industrial DED (200 - 3500 µm) and expand a larger range than Wolff et al.’s study ^3^. Our DED replicator can achieve multiple layers, traverse speed from 0.5 to 50 mm s^-1^, and laser power from 0 to 500 W, powder feed rate from 1 to 5 g min^-1^, which are also close to the industrial scales. The powder velocity from 2 to 10 m s^-1^ in our DED study is also in the industrial range. Our X-ray imaging results also show the conduction mode to match the industrial DED rather than the keyhole mode in the previous work ^3^. The energy density of the DED process in this work is nearly 125 - 500 J mm^-2^ with an available range of 0 - 1250 J mm^-2^, which covers the range of energy density of industrial DED processes with 4 - 189 J mm^-2^, and also shows a larger range than Wolff et al.’s study ^3^. This demonstrates that the mechanisms observed in this work can be applied to the industrial-scale DED. The laser spot size of 400 µm is also close to the industrial scale, although it is at the lower end of the range. One reason for choosing these parameters is also to achieve the optimal DED build and X-ray imaging observation quality to compromise the synchrotron X-ray beam.

## Supplementary Discussion 2

The force balance onto the bubble is measured by calculating the static buoyancy, the shear force by the molten metal flow and the pressure force by the molten metal flow. They are given as

Static buoyancy: $\int_{V} (\rho_{l}-\rho_{g})g\vec{n}_{z}dV$ (12)

Shear force on the surface: $\int_{A} \tau\vec{n}_{t}dA=\int_{A} \mu_{l}\left( \frac{\partial\left| \vec{u}_{//} \right|}{\partial n} \right)\vec{n}_{t}dA$ (13)

Pressure force on the surface (toward the bubble centre): $-\int_{A} p\vec{n}_{n}dA$ (14)

where $\rho_{l}$ is the molten metal density, $\rho_{g}$ is the gas density, *g* is the gravitational acceleration (= 9.8 m s^-2^), *V* is the bubble volume, *A* is the bubble surface area, $\mu_{l}$ is the molten metal viscosity, $\vec{u}_{//}=\vec{u}-(\vec{n}_{n}\cdot\vec{u})\vec{n}_{n}$ is the tangential flow velocity vector just above the bubble surface, $\vec{u}$ is the flow velocity and *n* represents the normal direction outward of the bubble. $\vec{n}_{z}=(0,0,1)$ is the unit vector pointing vertically upward, $\vec{n}_{n}={\nabla\rho}/\left| \nabla\rho\right|$ is the unit surface-normal vector pointing outward of the bubble and $\vec{n}_{t}=\left( \vec{u}-(\vec{n}_{n}\cdot\vec{u})\vec{n}_{n} \right)/\left| \vec{u}-(\vec{n}_{n}\cdot\vec{u})\vec{n}_{n} \right|$ is the unit surface-tangential vector (projected along with the flow velocity direction).

From the simulation result, the forces are calculated as shown in Supplementary Table 3. The force direction and magnitude are illustrated in Supplementary Fig. 12c, d, g and h and Supplementary Fig. 13c and d. The main flow effects are shown as the blue arrows.

In the case of “Large bubble pushed” (Fig. 7b), the large horizontal shear force above the bubble is mainly pushing the bubble in the horizontally backward (-*x*) direction (the blue arrow in Supplementary Fig. 12c). At the same time, the transverse Marangoni flow (the blue arrow in Supplementary Fig. 12d) pushes the bubble vertically downward (-*z*). The positive shear force in the +*z* direction is mainly due to the rising flow in the +*x* side of the bubble (while the local shear force is toward the –*z* direction in the –*x* side of the bubble), but this is minor. In total, the obliquely descending flow above the bubble surpasses the bubble rising effect due to buoyancy. This strong flow strip above the bubble is due to the Marangoni effect on the melt pool surface, which supplies energy (work) to the flow. Therefore, the bubble is pushed backward and downward when this flow structure is formed. It should be also noted that the shear force causes the bubble to rotate in the clockwise direction as schematically shown in Supplementary Fig. 12. This bubble rotation can also be confirmed in Supplementary Movie 2.

Note that the flow is unsteady and the quantitative magnitude of the forces may vary temporally, but the basic mechanism of pushing the bubble remains for some time. At 4.5 ms later (Supplementary Fig. 12g and h), the force analysis indicates that the bubble is still pushed by the flow in the same direction. At this time, the bottom of the bubble is in the solidifying front region and the bubble is captured and will remain inside the melt pool.

In the case of “Large bubble pop up” (Fig. 7c), due to the different locations where the bubble exists, the flow above the bubble is thinner and the shear force in the horizontally backward (-*x*) direction is weaker (the blue arrow in Supplementary Fig. 13c) and rather the front side flow pushes the bubble backward. More evidently, the transverse Marangoni cell flow (the blue arrow in Supplementary Fig. 13d) is much stronger at this *x* position (close to the melt pool centre in the *x* direction) and pushes the bubble vertically upward (+*z*) mostly by the shear force. Therefore, combined with the buoyancy, and with the fact that the distance between the bubble and the surface is closer, the bubble soon pops up to the melt pool surface and ruptures.

From the above results, it can be said that the structure of the Marangoni flow cells and the relative location of the bubble are the important factors to determine the bubble dynamics.

## Supplementary Tables

Supplementary Table 1. Material composition of RR1000 powder

| Nickel | Copper | Chromium | Cobalt | Molybdenum | Aluminium | Tantalum | Hafnium | Manganese |
| --- | --- | --- | --- | --- | --- | --- | --- | --- |
| 50 - 68% | < 0.5% | 10 - 22% | 0 - 20% | 0 - 10% | 0 - 5% | 0 - 2% | 0 - 1% | < 0.5% |

Supplementary Table 2. Comparison of key DED parameters between our *in situ* DED study with Wolff et al. ^3^ and industrial DED

|  | This in situ DED study | Wolff et al.’s study [^3^] | Industrial DED |
| --- | --- | --- | --- |
| Melt pool length (µm) | 500 - 2000 | 950 | 200 - 3500 [^4–6^] |
| Melt pool depth (µm) | 300 - 1200 | 280 | 200 - 2000 [^4–6^] |
| Track, layer # | 1 - 3 (1- n) * | 1 | n [^7–14^] |
| Traverse speed (mm s^-1^) | 1 - 2 (0.5 - 50) * | 100 | 5 - 28 [^7–14^] |
| Laser power (W) | 100 - 200 (0 - 500) * | 156, 208 | 270 - 5000 [^7–14^] |
| Laser spot size (µm) | 400 (100 - 700) * | 100 | 400 - 5000 [^7,9–14^] |
| Powder feed rate (g min^-1^) | 1.8 - 2.7 (1 - 5) * | 0.6 | 1 - 30 [^7–13^] |
| Linear energy density (J mm^-2^) | 125 - 500 (0 - 1250) * | 20.8 | 4 - 189 [^7–14^] |
| Powder shape | Spherical | Irregular | Spherical preferred [^12,13,15,16^] |
| Powder particle size (µm) | 53 - 106 (30 - 150) * | 75 - 200 | 30 - 150 [^12,13,15,16^] |
| Powder velocity (m s^-1^) | 2 - 10 | 1.5 - 3.2 | 2 - 30 [^17–19^] |

* Values before the bracket are those used for the runs in this study. Values in brackets are the range available with BAMPR II.

Supplementary Table 3. Force magnitude on the large bubble

| Case |  | +*x* direction (horizontally forward) | +*y* direction  (toward the centre of the melt pool) | +*z* direction  (vertically upward) |
| --- | --- | --- | --- | --- |
| Large bubble pushed | Buoyancy | - | - | 1.50×10^-7^ N |
|  | Shear force | -5.48×10^-7^ N | 0.29×10^-7^ N | 1.15×10^-7^ N |
|  | Pressure force | -0.16×10^-7^ N | -2.41×10^-7^ N | -7.21×10^-7^ N |
|  | Total force | -5.64×10^-7^ N | -2.12×10^-7^ N | -4.56×10^-7^ N |
| Large bubble pushed (4.5 ms later) | Buoyancy | - | - | 1.40×10^-7^ N |
|  | Shear force | -4.72×10^-7^ N | 1.47×10^-7^ N | 3.14×10^-7^ N |
|  | Pressure force | -2.78×10^-7^ N | -6.81×10^-7^ N | -5.93×10^-7^ N |
|  | Total force | -7.49×10^-7^ N | -5.33×10^-7^ N | -2.79×10^-7^ N |
| Large bubble pop up | Buoyancy | - | - | 1.48×10^-7^ N |
|  | Shear force | -0.49×10^-7^ N | -1.75×10^-7^ N | 6.37×10^-7^ N |
|  | Pressure force | -8.35×10^-7^ N | -2.58×10^-7^ N | 0.69×10^-7^ N |
|  | Total force | -8.84×10^-7^ N | -4.33×10^-7^ N | 7.06×10^-7^ N |

## Supplementary References

1. Shinjo, J. & Panwisawas, C. Chemical species mixing during direct energy deposition of bimetallic systems using titanium and dissimilar refractory metals for repair and biomedical applications. *Addit. Manuf.* **51**, 102654 (2022).

2. Panwisawas, C. *et al.* Additive manufacturability of superalloys: Process-induced porosity, cooling rate and metal vapour. *Addit. Manuf.* **47**, 102339 (2021).

3. Wolff, S. J. *et al.* In situ X-ray imaging of pore formation mechanisms and dynamics in laser powder-blown directed energy deposition additive manufacturing. *Int. J. Mach. Tools Manuf.* **166**, (2021).

4. Zhu, X. *et al.* Prediction of melt pool shape in additive manufacturing based on machine learning methods. *Opt. Laser Technol.* **159**, 108964 (2023).

5. Zhang, Y. M., Lim, C. W. J., Tang, C. & Li, B. Numerical investigation on heat transfer of melt pool and clad generation in directed energy deposition of stainless steel. *Int. J. Therm. Sci.* **165**, (2021).

6. Zhang, P. *et al.* Effects of melt-pool geometry on the oriented to misoriented transition in directed energy deposition of a single-crystal superalloy. *Addit. Manuf.* **60**, 103253 (2022).

7. Amine, T., Newkirk, J. W. & Liou, F. Investigation of effect of process parameters on multilayer builds by direct metal deposition. *Appl. Therm. Eng.* **73**, 500–511 (2014).

8. Ma, M., Wang, Z. & Zeng, X. A comparison on metallurgical behaviors of 316L stainless steel by selective laser melting and laser cladding deposition. *Mater. Sci. Eng. A* **685**, 265–273 (2017).

9. Song, J. *et al.* Numerical and experimental study of laser aided additive manufacturing for melt-pool profile and grain orientation analysis. *Mater. Des.* **137**, 286–297 (2018).

10. Zhai, Y., Galarraga, H. & Lados, D. A. Microstructure, static properties, and fatigue crack growth mechanisms in Ti-6Al-4V fabricated by additive manufacturing: LENS and EBM. *Eng. Fail. Anal.* **69**, 3–14 (2016).

11. Fang, J. X. *et al.* The effects of solid-state phase transformation upon stress evolution in laser metal powder deposition. *Mater. Des.* **87**, 807–814 (2015).

12. Kong, Y., Zhao, L., Zhu, L. & Huang, H. The selection of laser beam diameter in directed energy deposition of austenitic stainless steel: A comprehensive assessment. *Addit. Manuf.* **52**, 102646 (2022).

13. Kies, F. *et al.* Defect formation and prevention in directed energy deposition of high-manganese steels and the effect on mechanical properties. *Mater. Sci. Eng. A* **772**, 138688 (2020).

14. Liu, Y. *et al.* Microstructure and mechanical behavior of additively manufactured CoCrFeMnNi high-entropy alloys: Laser directed energy deposition versus powder bed fusion. *Acta Mater.* **250**, (2023).

15. Anderson, I. E., White, E. M. H. & Dehoff, R. Feedstock powder processing research needs for additive manufacturing development. *Curr. Opin. Solid State Mater. Sci.* **22**, 8–15 (2018).

16. Ahn, D. G. *Directed Energy Deposition (DED) Process: State of the Art*. *International Journal of Precision Engineering and Manufacturing - Green Technology* vol. 8 (Korean Society for Precision Engineering, 2021).

17. Balu, P., Leggett, P. & Kovacevic, R. Parametric study on a coaxial multi-material powder flow in laser-based powder deposition process. *J. Mater. Process. Technol.* **212**, 1598–1610 (2012).

18. Ibarra-Medina, J. & Pinkerton, A. J. Numerical investigation of powder heating in coaxial laser metal deposition. *Surf. Eng.* **27**, 754–761 (2011).

19. Kovalev, O. B., Zaitsev, A. V., Novichenko, D. & Smurov, I. Theoretical and experimental investigation of gas flows, powder transport and heating in coaxial laser direct metal deposition (DMD) process. *J. Therm. Spray Technol.* **20**, 465–478 (2011).
