# Supplementary material for: Pore evolution mechanisms during directed energy deposition additive manufacturing
Source: Nat Commun. 2024 Feb 24;15:1715. doi: 10.1038/s41467-024-45913-9 (PMC10894260; doi:10.1038/s41467-024-45913-9)
Supplement: Supplementary file 3 — Description of Additional Supplementary Information [file 41467_2024_45913_MOESM3_ESM.docx]

## Description of Supplementary Movies

## File name: Supplementary Movie 1

Description: Pore evolution during the DED process of RR1000 nickel-based superalloy in the synchrotron X-ray radiography with a Dimax camera. The camera frame rate is 1 kHz, and the pixel size is 3.7 µm. The laser power is 160 W, the traverse speed is 2 mm s^-1^, layer 3.

## File name: Supplementary Movie 2

Description: Pore evolution during the DED process of RR1000 nickel-based superalloy in the synchrotron X-ray radiography with a Photron camera. The camera frame rate is 20 kHz, and the pixel size is 4 µm. The laser power is 160 W, the traverse speed is 2 mm s^-1^, layer 3.

## File name: Supplementary Movie 3

Description: Pore evolution during the DED process of RR1000 nickel-based superalloy in the synchrotron X-ray radiography with a Photron camera. The camera frame rate is 20 kHz, and the pixel size is 4 µm. The laser power is 160 W, the traverse speed is 1 mm s^-1^, layer 1.

## File name: Supplementary Movie 4

Description: Pore evolution during the DED process of RR1000 nickel-based superalloy in the synchrotron X-ray radiography with a Dimax camera. The camera frame rate is 1 kHz, and the pixel size is 3.7 µm. The laser power is 160 W, the traverse speed is 1 mm s^-1^, layer 3.

## File name: Supplementary Movie 5

Description: Pore evolution during the DED process of RR1000 nickel-based superalloy in the synchrotron X-ray radiography with a Photron camera. With bubble tracking. The camera frame rate is 20 kHz, and the pixel size is 4 µm. The laser power is 160 W, the traverse speed is 2 mm s^-1^, layer 3.

## File name: Supplementary Movie 6

Description: Pore evolution during the DED process of RR1000 nickel-based superalloy in the synchrotron X-ray radiography with a Photron camera. With bubble tracking. The camera frame rate is 20 kHz, and the pixel size is 4 µm. The laser power is 160 W, the traverse speed is 1 mm s^-1^, layer 3.

## File name: Supplementary Movie 7

Description: Multiphysics modelling results showing bubble coalescence in the rear/back location of the melt pool. The laser power is 160 W, and the traverse speed is 2 mm s^-1^.

## File name: Supplementary Movie 8

Description: Multiphysics modelling results showing bubble coalescence in the front-deep location of the melt pool. The laser power is 160 W, and the traverse speed is 2 mm s^-1^.

## File name: Supplementary Movie 9

Description: Multiphysics modelling results showing bubble pushing in the rear/back location of the melt pool. The laser power is 160 W, and the traverse speed is 2 mm s^-1^.
